# Supplementary material for: Genome-wide identification and phenotypic characterization of seizure-associated copy number variations in 741,075 individuals
Source: Nat Commun. 2023 Jul 20;14:4392. doi: 10.1038/s41467-023-39539-6 (PMC10359300; doi:10.1038/s41467-023-39539-6)
Supplement: Supplementary file 1 — Supplementary Information [file 41467_2023_39539_MOESM1_ESM.pdf]

Genome-wide identification and phenotypic characterization of seizure-associated copy number variations in 741,075 individuals.

SUPPLEMENTARY MATERIAL

SUPPLEMENTARY TABLES

Supplementary Table 1: List of cohorts for which CNV calls were made.

Cohort	Data Source	Short Name	Citation	Original Ref. Build	Case Phenotypes	Cases	Controls	Meta-analysis cohort assignment
**Epi25 Collaborative cohort**	Epi25 Collaborative	Epi25	Epi25 Collaborative, Am J Hum Genet (2021)	hg19	Epilepsy and controls	16,109	8,545	NA
**Neuropsychiatric cohort**	BioVU	BioVU	Morley et al., Nat. Med. (2021)	hg19	Mixed	32,306	14,661	Cohort 7
	Boston Children's Hospital	BCH	Talkowski et al., Cell (2012)	hg18	Mixed	3,591	0	Cohort 1
	Children's Hospital of Philadelphia	CHOP	Li et al., Nat. Commun. (2020)	hg19	Mixed	153,870	24,161	Cohort 4
	Eichler Lab	Coe	Coe et al., Nat. Genet. (2014)	hg19	Developmental disorders	29,104	11,256	Cohort 1
	Eichler Lab	Cooper	Cooper et al., Nat. Genet. (2011)	hg19	N/A	0	8,329	Cohort 1
	Estonian Biobank	EstBB	Leitsalu et al., Int. J. Epidemiol. (2014)	hg19	Mixed	63,183	15,659	Cohort 6
	GeneDX	GDX	-	hg18 &19	Mixed	74,028	0	Cohort 2
	Indiana University	IU	-	hg19	Mixed	1,577	0	Cohort 1
	Ontario Population Genomics Platform	Ontario	Uddin et al., Genet. Med. (2015)	hg19	N/A	0	873	Cohort 2
	Psychiatric Genetics Consortium	PGC	Marshall et al., Nat. Genet. (2017)	hg18	Schizophrenia	21,094	20,277	Cohort 3
	Radboud University Medical Center	RUMC	Vulto-van Silfhout et al., Hum. Mutat. (2013)	hg17	Intellectual disability	5,531	0	Cohort 3
	SickKids Hospital	SickKids	Zarrei et al., NPJ Genomic Medicine (2019)	hg19	Developmental disorders	2,689	0	Cohort 3
	Simons Simplex Collection	SSC	Sanders et al., Neuron (2015)	hg18	Autism	2,795	0	Cohort 3
	The Cancer Genome Atlas	TCGA	Zack et al., Nat. Genet. (2013)	hg19	N/A	0	8,670	Cohort 3
	The Genetic Etiology of Tourette Syndrome Consortium	TSAICG	Huang et al., Neuron (2017)	hg19	Tourette Syndrome	2,434	4,093	Cohort 2
	UK Biobank	UKBB	Macé et al., Nat. Commun. (2017)	hg19	Mixed	54,071	375,800	Cohort 2 (3,139 randomly selected controls); Cohort 5 (all remaining samples)
**Phenomic Cohort**	Epi25 Collaborative	Pheno-Epi25	Epi25 Collaborative, Am J Hum Genet (2021)	hg19	Non-acquired epilepsy	10,880	0	NA

The phenomic cohort is a subset of the Epi25 cohort.

Supplementary Table 2: Phenotypes of neuropsychiatric disorders cohorts used in this study.

Disorder Full Name	N	HPO
Seizures	10,590	HP:0001250
**Considered 23 neuropsychiatric disorders**		
Abnormality of the nervous system	248,751	HP:0000707
Abnormality of higher mental function	15,416	HP:0011446
Neurodevelopmental abnormality	55,760	HP:0012759
Autistic behavior	26,199	HP:0000729
Intellectual disability	10,371	HP:0001249
Behavioral abnormality	135,483	HP;0000708
Impairment in personality functioning	58,323	HP:0031466
Hyperactivity	14,955	HP:0000752
Bipolar affective disorder	34,915	HP:0007302
Schizophrenia	25,108	HP:0100753
Abnormal fear/anxiety-related behavior	25,856	HP:0100852
Sleep disturbance	6,091	HP:0002360
Abnormality of nervous system morphology	68,594	HP:0012639
Morphological abnormality of the central nervous system	37,800	HP:0002011
Abnormality of brain morphology	7,688	HP:0012443
Atrophy/Degeneration affecting the central nervous system	26,163	HP:0007367
Abnormality of central motor function	5,341	HP:0011442
Abnormality of movement	12,628	HP:0100022
CNS hypermyelination	3,204	HP:0012447
Involuntary movements	4,928	HP:0004305
Abnormality of the peripheral nervous system	6,005	HP:0410008
Abnormal peripheral nervous system morphology	32,665	HP:0000759
Peripheral neuropathy	22,571	HP:0009830

In column 1, the Human Phenotype Ontology code of the considered disorder is reported along with the full name. In the last column the number of individuals with the specific disorder are reported.

Supplementary Table 3: The percentage of 10,880 Epi25 participants included in the phenomic analysis cohort annotated with a clinically-prioritized selection of common epilepsy phenotypes. The full list of phenotype frequencies is available in Supplementary Data 4.

HPO id	HPO name	Number of annotated individuals	Percentage of annotated individuals
HP:0001250	Seizure	10869	99.9
HP:0002353	EEG abnormality	9127	83.9
HP:0007359	Focal-onset seizure	6991	64.3
HP:0002069	Bilateral tonic-clonic seizure	5772	53.1
HP:0011146	Dialeptic seizure	5608	51.5
HP:0011185	EEG with focal epileptiform discharges	4987	45.8
HP:0033259	Non-motor seizure	3959	36.4
HP:0011198	EEG with generalized epileptiform discharges	3924	36.1
HP:0002384	Focal impaired awareness seizure	3866	35.5
HP:0002197	Generalized-onset seizure	3786	34.8
HP:0012443	Abnormality of brain morphology	3695	34.0
HP:0033717	EEG with temporal epileptiform discharges	3489	32.1
HP:0007334	Bilateral tonic-clonic seizure with focal onset	3168	29.1
HP:0032677	Generalized-onset motor seizure	2931	26.9
HP:0020174	Refractory drug response	2658	24.4
HP:0012759	Neurodevelopmental abnormality	1895	17.4

EEG = electroencephalogram.

Supplementary Table 4: CNV frequencies in the cases & controls of the meta-analysis, DGV-Gold Standard, and DECIPHER databases.

Cytoband	CNV type	Hg19 Start (Mb)	Hg19 End (Mb)	Cases carrier frequency [%]	Control carrier frequency [%]	Frequency in DGV Gold Standard [%]	Frequency in DECIPHER [%]
1p36.33	DEL	0.91	1.51	0.086	0.015	0	0
1p36.33	DEL	2.02	2.49	0.146	0.003	0	0
1q44	DEL	245.29	245.86	0.041	0.002	0	0
2p21-p16.3	DEL	47.5	47.85	0.678	0.002	0	0
2q13	DUP	110.77	111.06	0.139	0.108	0	0.27
3q29	DEL	195.76	196.24	0.034	0.002	0	0.12
8p23.3-p23.2	DEL	0.4	5.47	0.067	0.010	0	0
9p24.3	DEL	0.33	0.56	0.049	0.007	0	0.02
9q34.3	DUP	139.21	140.12	0.315	0.003	0	0
10q26.3	DEL	133.41	134.68	0.030	0.002	0	0
15q11.2	DEL	22.74	23.28	0.689	0.284	0.41	0.83
15q11.2-q13.3	DUP	22.98	32.15	0.258	0.005	0	0.12
15q12-q13.1	DEL	27.93	28.23	0.097	0.008	0	0
15q13.2-q13.3	DEL	31.06	32.51	0.243	0.012	0	0.02
16p13.3	DUP	0.6	0.89	0.573	0.009	0	0
16p13.11	DEL	15.42	16.35	0.363	0.031	0.03	0.07
16p12.2	DEL	21.88	22.5	0.191	0.054	0.09	0.08
16p11.2	DEL	29.56	30.19	0.165	0.024	0.05	0.12
16p11.2	DUP	29.87	30.19	0.127	0.026	0	0.03
17q12	DUP	34.76	36.25	0.187	0.014	0.02	0.12
17q21.31	DEL	41.08	41.45	0.461	0.004	0	0
19p13.3	DUP	1.04	1.34	0.427	0.003	0	0.08
20q13.33	DUP	62	62.35	0.479	0.006	0	0
22q11.21	DUP	18.99	21.54	0.199	0.067	0	0
22q11.21	DEL	18.99	21.54	0.120	0.009	0	0.19

The frequencies in the DGV Gold Standard and DECIPHER Population databases are given for CNVs with ≥50% overlap with the seizure-associated CNV regions.

Supplementary Table 5: Resources used to collect information about CNVs and the affected genes.

Name	Web address	Used for
DGV	http://dgv.tcag.ca/dgv/app/downloads?ref=	Annotation of CNV frequencies from external data
DECIPHER	https://www.deciphergenomics.org/about/downloads/data	Localization of reported phenotypes, curated genes, dosage sensitivity
	https://www.deciphergenomics.org	Reported CNV Syndromes
ClinGen	https://www.clinicalgenome.org	Localization of reported phenotypes, curated genes, dosage sensitivity
GARD	https://rarediseases.info.nih.gov	Detailed phenotypes of disorders caused by CNVs or genes overlapping with the seizure-associated regions
OMIM	https://www.omim.org	Molecular Genetics and reported patients for any gene
PubMed	https://pubmed.ncbi.nlm.nih.gov	Screening of published literature
UCSC Genome Browser on Human GRCh37/hg19	https://genome.ucsc.edu/	Visualization of the credible intervals
GWAS Catalog	https://www.ebi.ac.uk/gwas/home	Reported GWAS hits in credible intervals
Enrichr	https://maayanlab.cloud/Enrichr/	Pathway analyses

Supplementary Table 6: List of modifications to the HPO (version released 2022-02-14).

HPO id	HPO name	Rationale for exclusion or modified use
HP:0000001	All	Excluded as ubiquitous after propagation in this cohort.
HP:0000118	Phenotypic abnormality	Excluded as ubiquitous after propagation in this cohort.
HP:0000707	Abnormality of the nervous system	Excluded as ubiquitous after propagation in this cohort.
HP:0012638	Abnormal nervous system physiology	Excluded as ubiquitous after propagation in this cohort.
HP:0020174	Refractory drug response	Made child of Phenotypic abnormality [HP:0000118] only because in the context of this analysis, we do not consider this to be a form an Abnormality of metabolism/homeostasis [HP:0001939]. Abnormal drug response [HP:0020169] was excluded from this modification because no individual was annotated with this without also being annotated with Refractory drug response [HP:0020174], making the former uninformative relative to the latter.
HP:0033349	Seizure cluster	Made child of Seizure [HP:0001250] only as a description of a phenotypic feature of seizures.
HP:0040006	Mortality/aging	We used this term to code those individuals who were recorded as deceased.
HP:0001699	Sudden death	Made child of Mortality/Aging [HP:0040006] only, a term we used to code those individuals who were recorded as deceased. Sudden unexpected death in epilepsy [HP:0033258] remained a child of Sudden death [HP:0001699].
HP:0025142	Constitutional symptom	Only very few individuals were annotated with types of Constitutional symptom [HP:0025142] (Fatigue [HP:0012378] in two and Myalgia [HP:0003326] in one), which we consider to be a sign of gross underreporting given the frequency of these in the general population. Hence, we omitted the term Constitutional symptom [HP:0025142] to avoid overweighting of the similarity between what are phenotypically only loosely related symptoms, making Fatigue [HP:0012378] and Pain [HP:0012531] (parent of Myalgia [HP:0003326]) children of Phenotypic abnormality [HP:0000118] only.
HP:0002372	Normal interictal EEG	While the absence of electroencephalographic abnormalities can be characteristic of some epilepsies, particularly in their early stages, we excluded this term because in HPO release 2022-02-14 it is considered a type of Interictal EEG abnormality [HP:0025373] and we did not wish the presence of a normal EEG to imply an abnormal interictal EEG.

SUPPLEMENTARY FIGURES

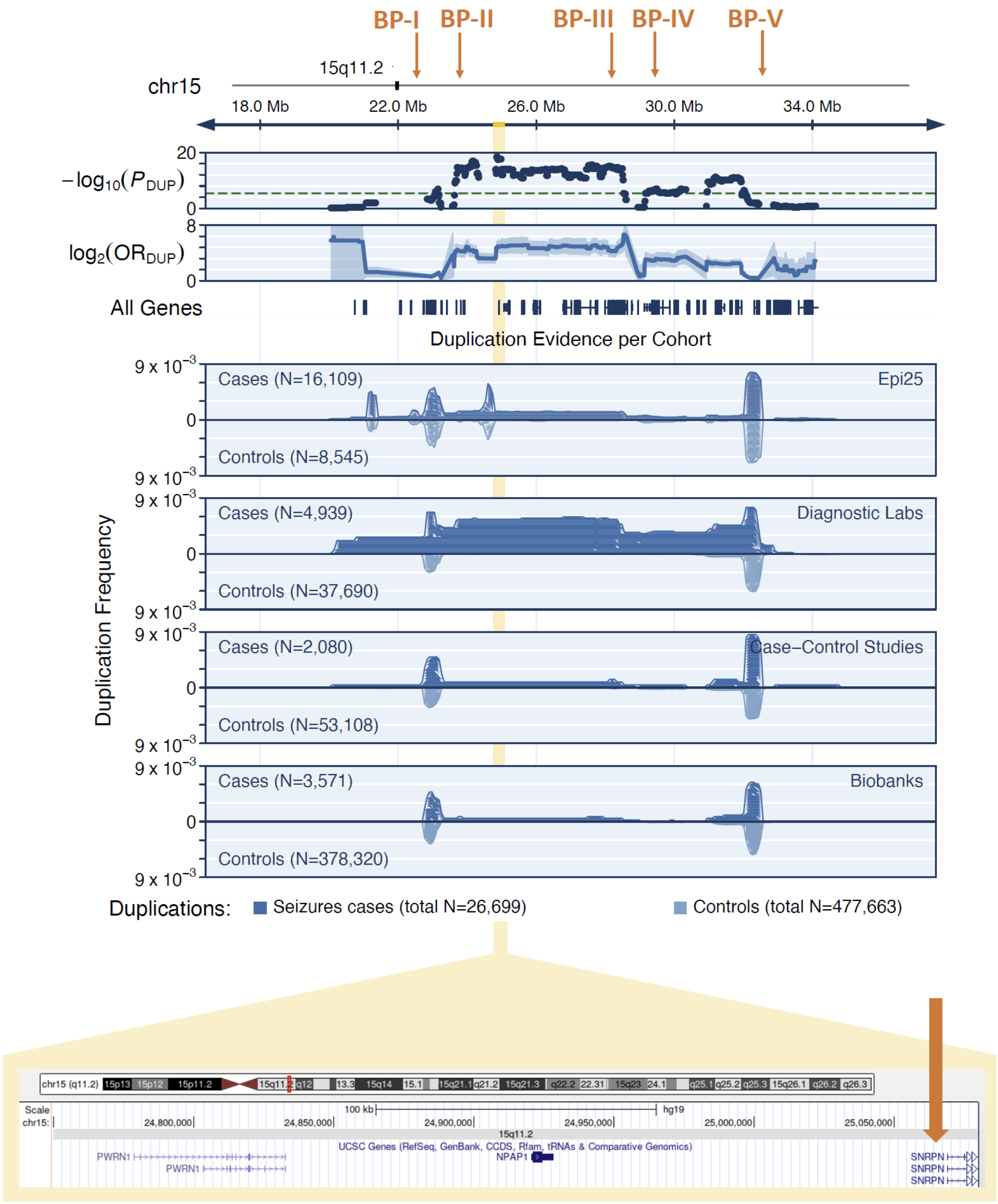

Supplementary Fig. 1: Regional CNV distribution plot and fine-mapping of the 15q11-q13 duplication. Known breakpoints are labeled BP-I to BP-V. The dashed line in the first plot represents the Bonferroni-corrected threshold for genome-wide significance, alpha=3.74x10^{-6}. The credible interval containing the causal element/gene with 95% confidence is highlighted in yellow. The dark orange arrow in the lower plot points to the candidate gene of the interval.

Supplementary Figure 2A)

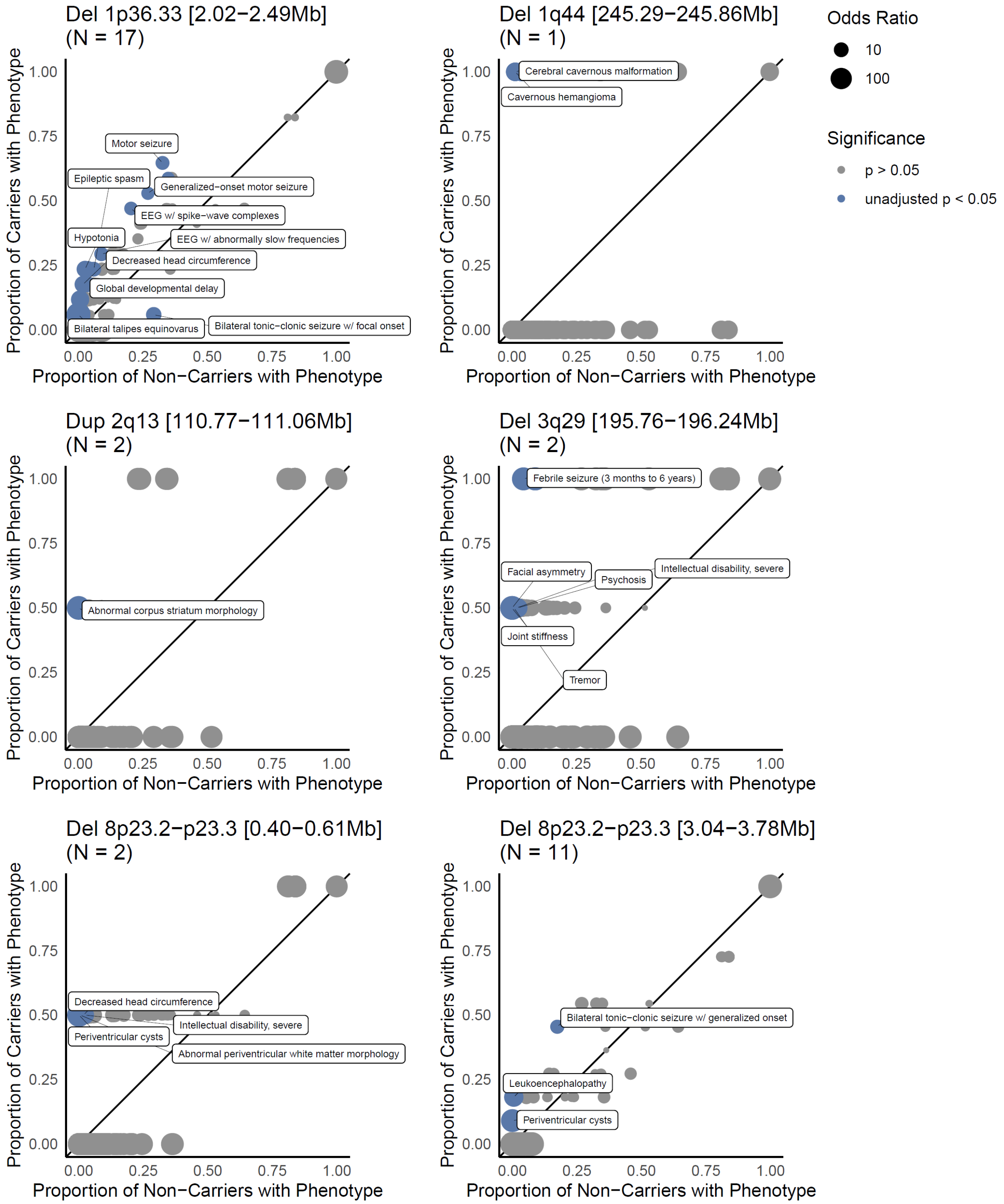

Supplementary Figure 2B)

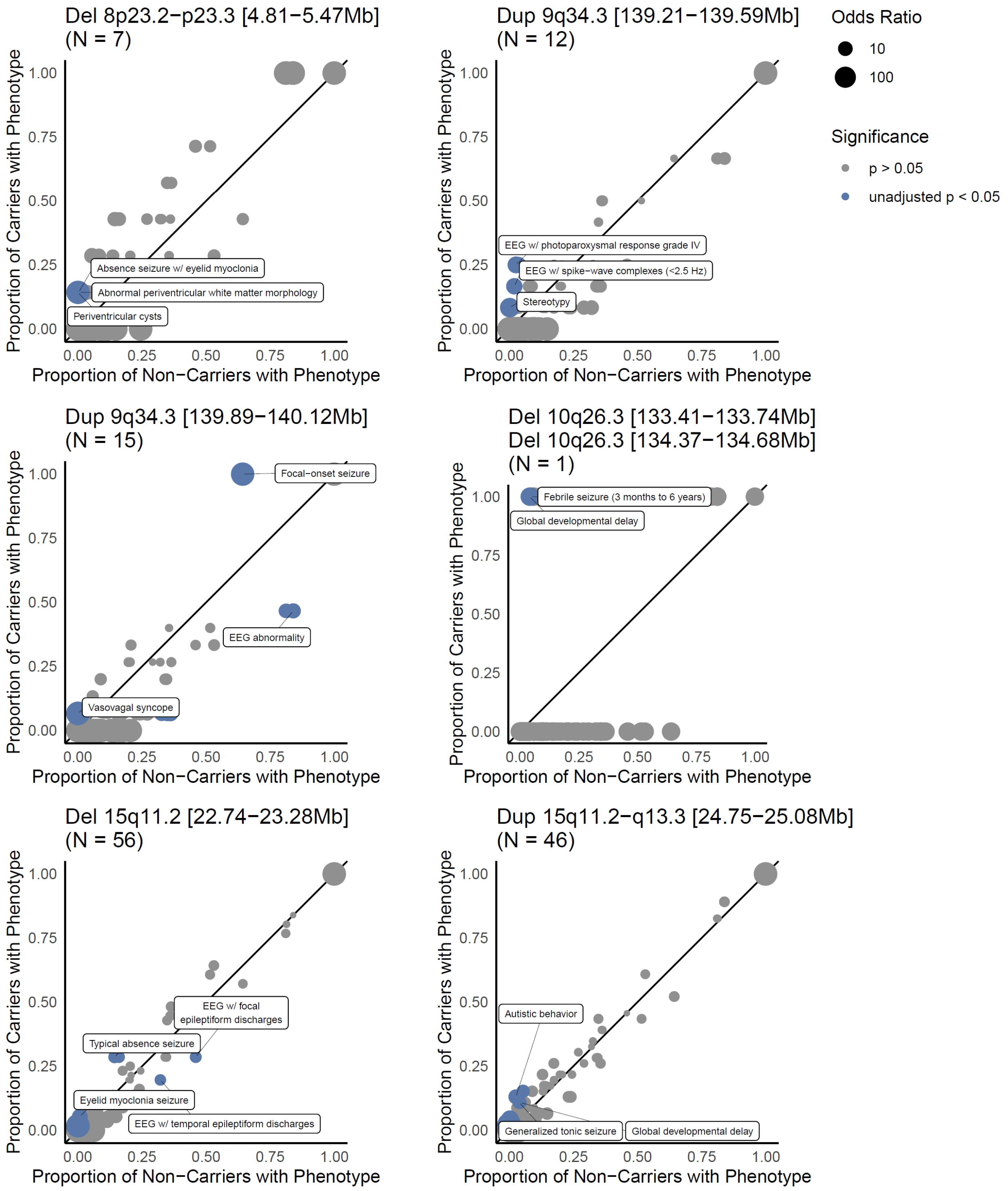

Supplementary Figure 2C)

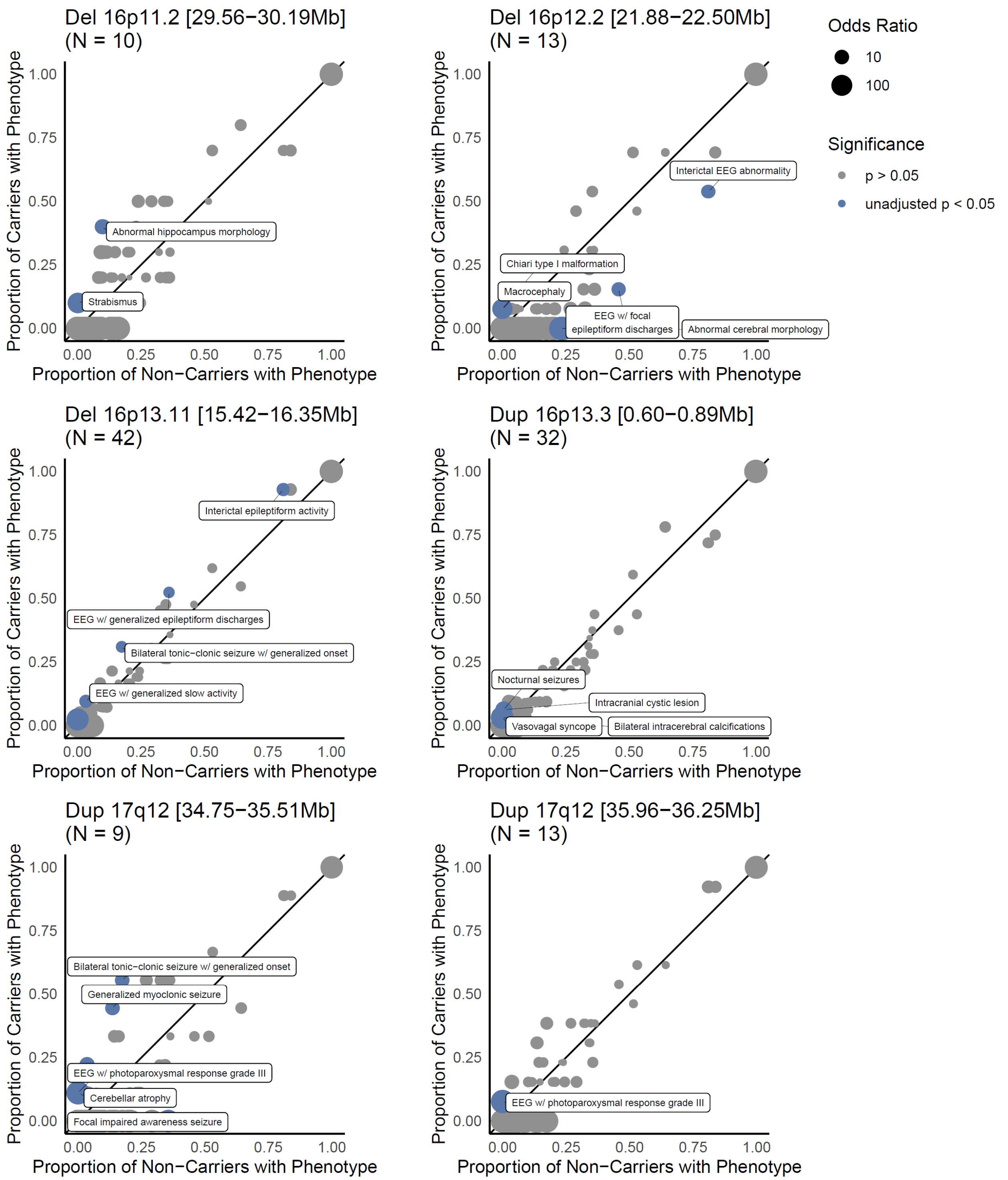

Supplementary Figure 2D)

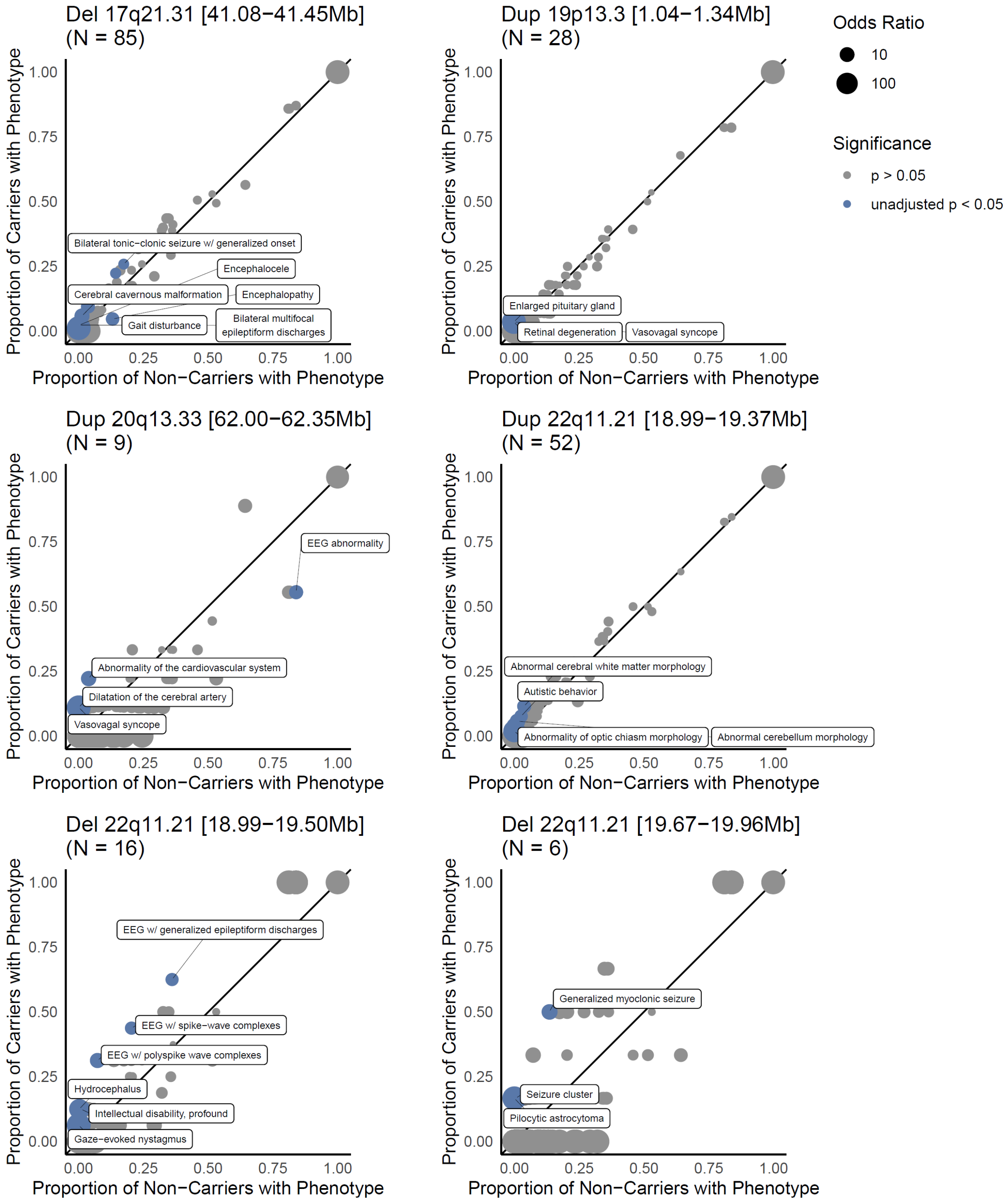

Supplementary Figure 2E)

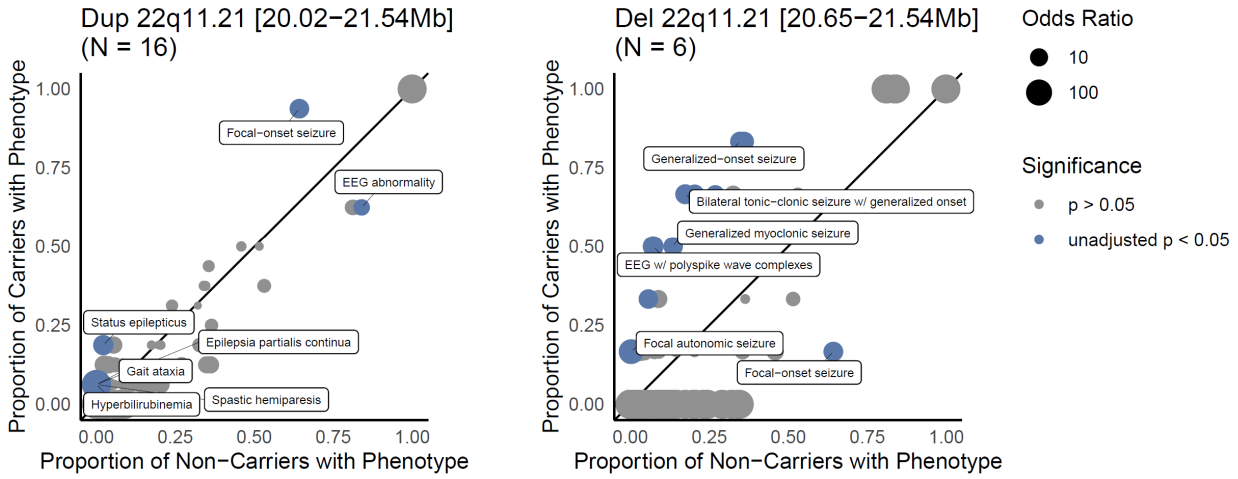

Supplementary Fig. 2A-E: The enrichment and depletion of phenotypes among 10,880 Epi25 participants 60 carrying CNVs (complements Figure 2 of the main manuscript). For each CNV, the proportion of carriers and non-carriers annotated with each HPO concept is plotted. Those above the diagonal were enriched among carriers, and those below were depleted. Odds ratios are represented by dot size. The selected phenotypes labeled were prioritized according to statistical evidence and clinical breadth. Full results for all associations reaching unadjusted *P*<0.05 are provided in **Supplementary Table 6**. EEG = electroencephalogram, w/ = with.

Supplementary Figure 3A)

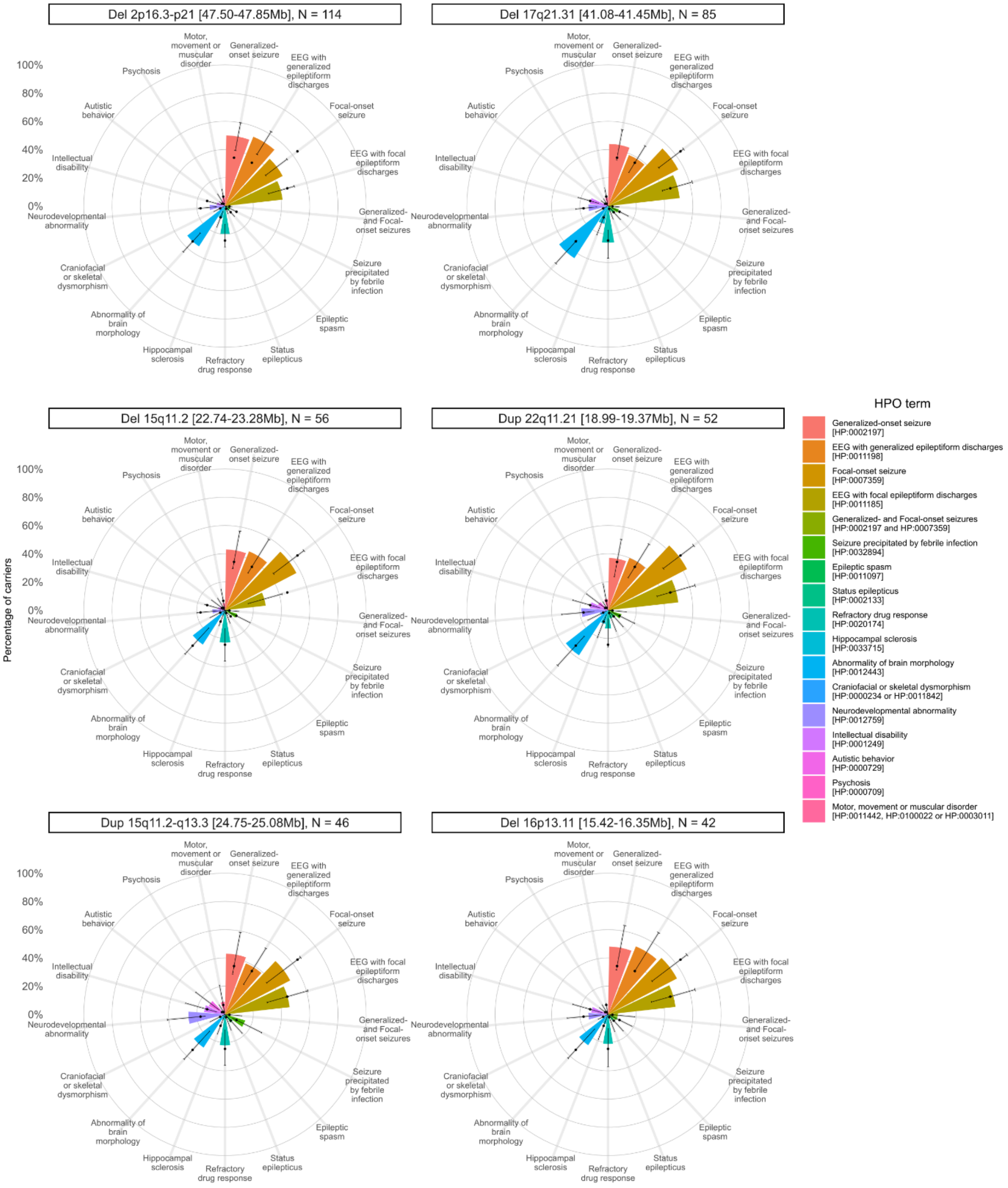

Supplementary Figure 3B)

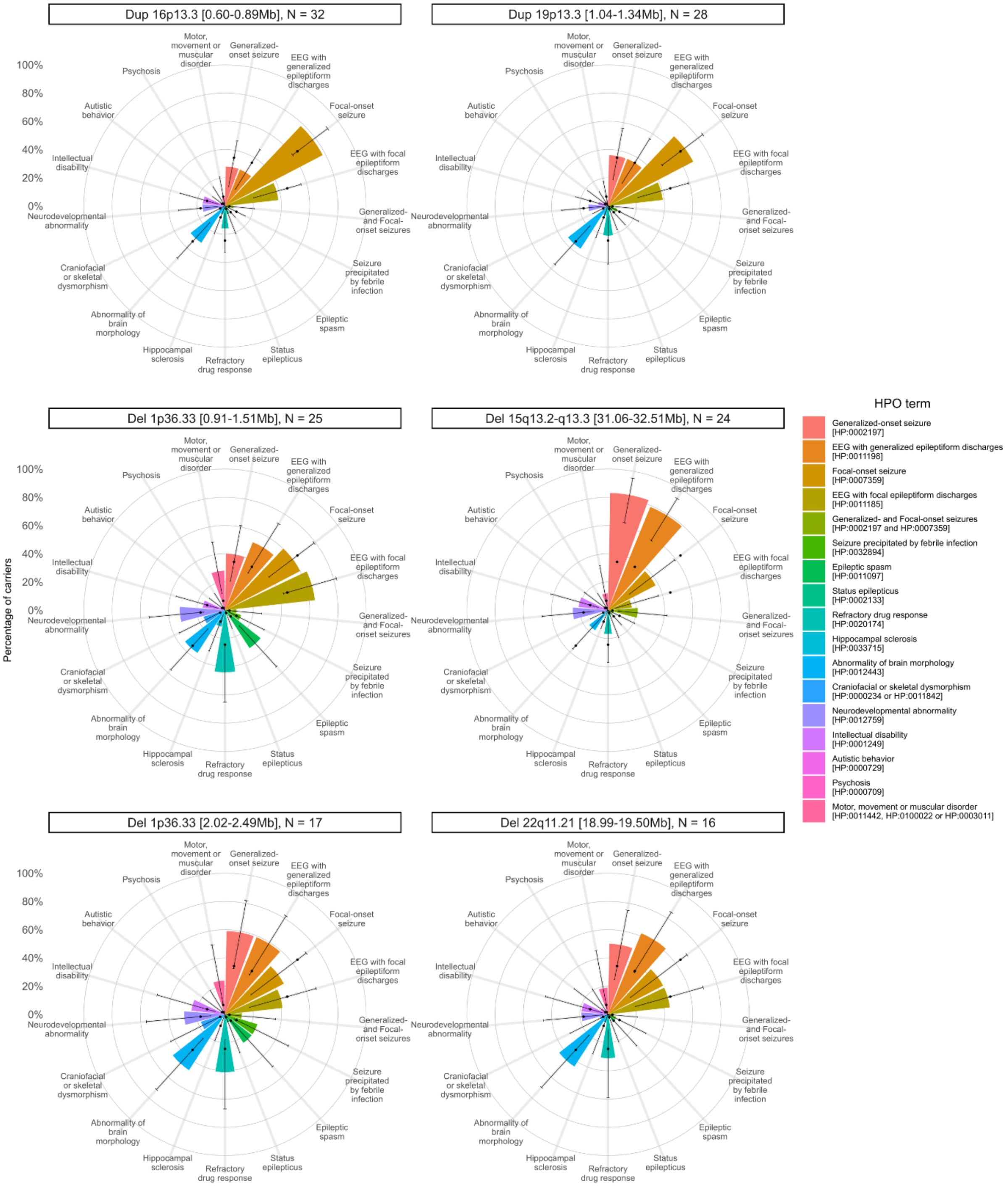

Supplementary Figure 3C)

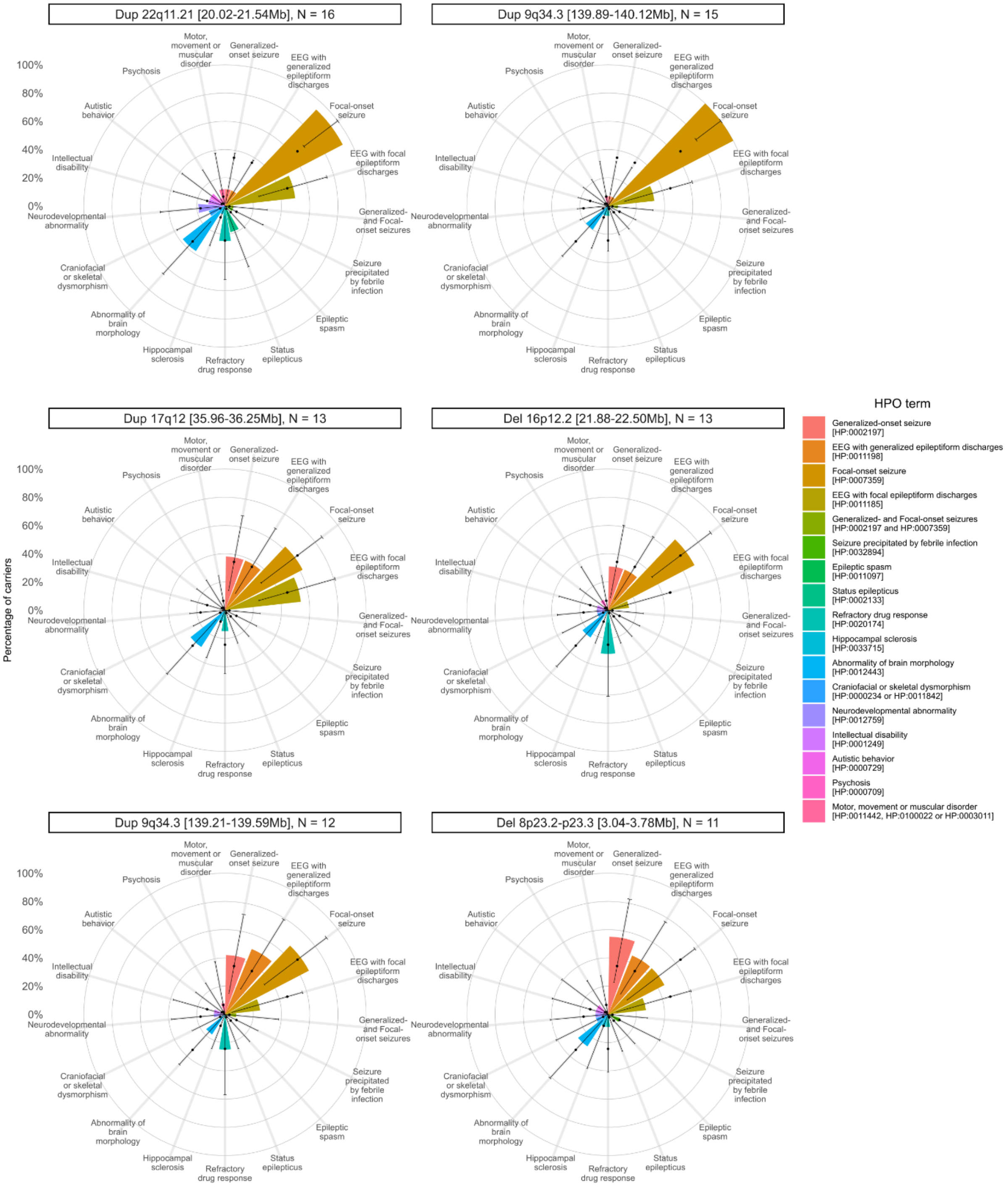

Supplementary Figure 3D)

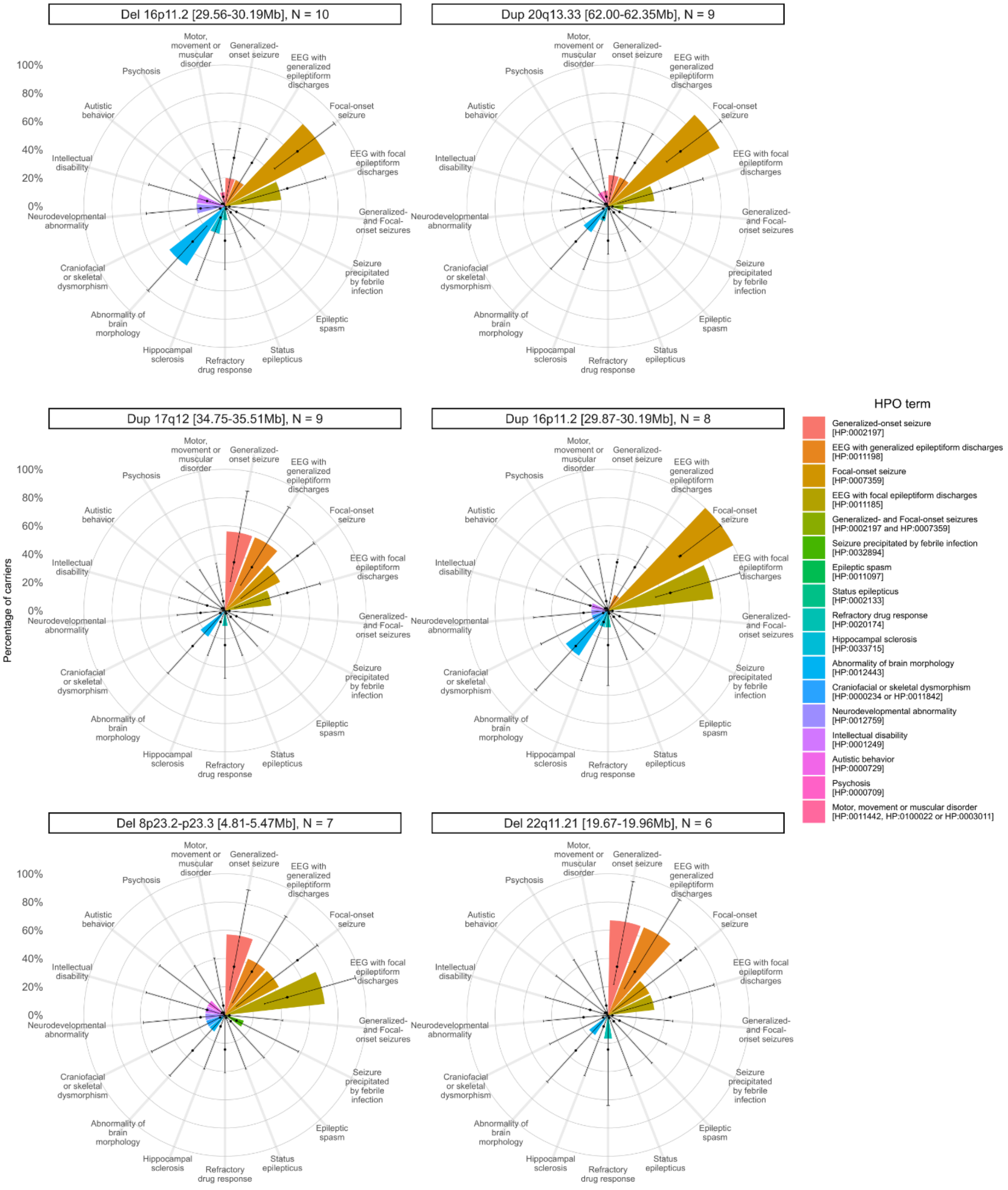

Supplementary Figure 3E)

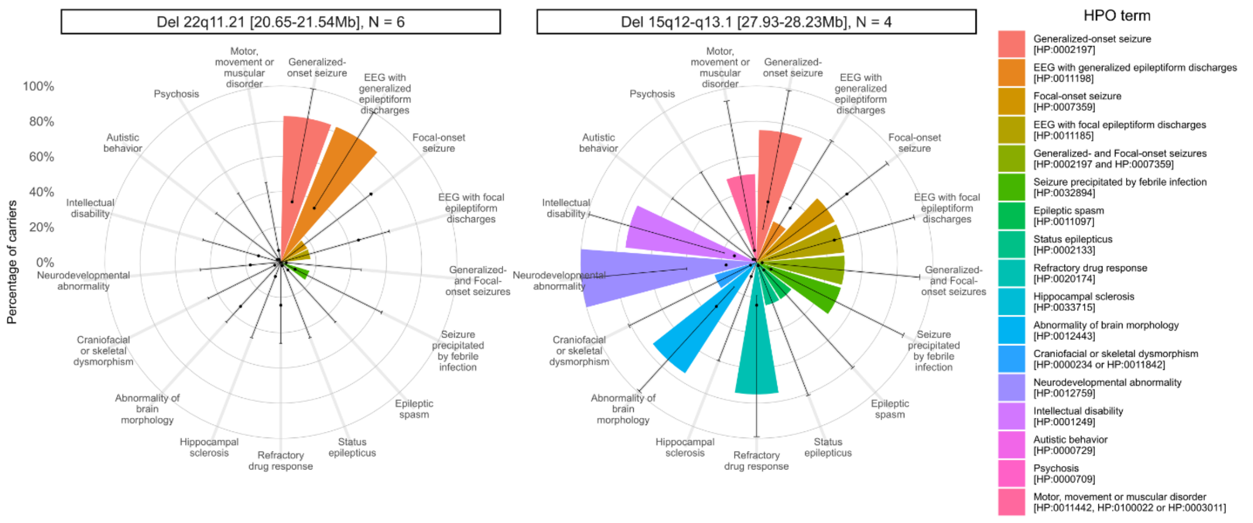

Supplementary Figures 3A-E: Summaries of the clinical features of carriers of each CNV in the cohort of 64 10,880 Epi25 participants (including the panels from Fig, 3 of the main manuscript). The percentage of carriers of the CNV with each broad phenotype is shown by the height of bars arranged on a polar axis, with two-sided 95% confidence interval error bars for these percentages derived from the binomial distribution using *stats::binom.test()*. For reference, dots indicate the percentage of the entire Phenomic cohort of 10,880 people with each broad phenotype (representing the prior probability of a person having the phenotype without genetic stratification). The binomial distribution two-sided 95% confidence intervals for a cohort size of 10,880 are no wider than 1.9% (not shown for clarity). "Craniofacial or skeletal dysmorphism" includes individuals with either “Abnormality of the head [HP:0000234]” (which excludes isolated brain structural abnormalities) or “Abnormal skeletal morphology [HP:0011842]”. “Motor, movement or muscular disorder” includes individuals with any of “Abnormal central motor function [HP:0011442]”, “Abnormality of movement [HP:0100022]” or “Abnormality of the musculature [HP:0003011]”, but not “Motor delay [HP:0001270]”, which is included in “Neurodevelopmental abnormality”. While “Neurodevelopmental abnormality” includes those with “Intellectual disability”, the latter is shown additionally as it is a neurodevelopmental outcome with particularly important socioeconomically important consequences. EEG = electroencephalogram.
