# Supplementary material for: Genome-wide identification and phenotypic characterization of seizure-associated copy number variations in 741,075 individuals
Source: Nat Commun. 2023 Jul 20;14:4392. doi: 10.1038/s41467-023-39539-6 (PMC10359300; doi:10.1038/s41467-023-39539-6)
Supplement: Supplementary file 3 — Description of Additional Supplementary Files [file 41467_2023_39539_MOESM3_ESM.pdf]

Description of Additional Supplementary Files

File Name: Supplementary Data 1
Description: List of candidate genes of lower confidence within the identified credible intervals.

File Name: Supplementary Data 2
Description: All genes inside the credible intervals are reported.

File Name: Supplementary Data 3
Description: All CNV-HPO associations with unadjusted two-sided $P<0.05$.

File Name: Supplementary Data 4
Description: The proportion of 10,880 Epi25 participants annotated with each HPO concept after propagation.
