# Supplementary material for: Genome-wide identification and phenotypic characterization of seizure-associated copy number variations in 741,075 individuals
Source: Nat Commun. 2023 Jul 20;14:4392. doi: 10.1038/s41467-023-39539-6 (PMC10359300; doi:10.1038/s41467-023-39539-6)
Supplement: Supplementary file 8 — Reporting Summary [file 41467_2023_39539_MOESM8_ESM.pdf]

nature portfolio

Corresponding author(s): Costin Leu, Dennis Lal

Last updated by author(s): 2023/05/08

Reporting Summary

Nature Portfolio wishes to improve the reproducibility of the work that we publish. This form provides structure for consistency and transparency in reporting. For further information on Nature Portfolio policies, see our Editorial Policies and the Editorial Policy Checklist.

Statistics

For all statistical analyses, confirm that the following items are present in the figure legend, table legend, main text, or Methods section.

n/a	Confirmed	
☐	☒	The exact sample size (*n*) for each experimental group/condition, given as a discrete number and unit of measurement
☐	☒	A statement on whether measurements were taken from distinct samples or whether the same sample was measured repeatedly
☐	☒	The statistical test(s) used AND whether they are one- or two-sided<br>*Only common tests should be described solely by name; describe more complex techniques in the Methods section.*
☒	☐	A description of all covariates tested
☐	☒	A description of any assumptions or corrections, such as tests of normality and adjustment for multiple comparisons
☐	☒	A full description of the statistical parameters including central tendency (e.g. means) or other basic estimates (e.g. regression coefficient) AND variation (e.g. standard deviation) or associated estimates of uncertainty (e.g. confidence intervals)
☐	☒	For null hypothesis testing, the test statistic (e.g. *F*, *t*, *r*) with confidence intervals, effect sizes, degrees of freedom and *P* value noted<br>*Give P values as exact values whenever suitable.*
☒	☐	For Bayesian analysis, information on the choice of priors and Markov chain Monte Carlo settings
☒	☐	For hierarchical and complex designs, identification of the appropriate level for tests and full reporting of outcomes
☒	☐	Estimates of effect sizes (e.g. Cohen's *d*, Pearson's *r*), indicating how they were calculated

Our web collection on statistics for biologists contains articles on many of the points above.

Software and code

Policy information about availability of computer code

Data collection	No software was used to collect data. The raw genotyping data were stored and analyzed on the same HPC Linux server. Summary statistics from the seizure and neuropsychiatric disorders cohort (Collins et al., 2022) were exchanged through a Google Cloud storage bucket.
Data analysis	We used the following software for 1) Genotyping QC and CNV calling: Plink v1.9, PennCNV. 2) Association analyses: custom R (v.3.6.1) and Python (v.3.7.9) scripts available on GitHub (https://github.com/talkowski-lab/rCNV2) and deposited at Zenodo (https://github.com/talkowski-lab/rCNV2/tree/v1.0, with DOI 10.5281/zenodo.6647918). metaphor R (v3.6.1) package for meta-analyses; 3) HPO-based association analysis – several R packages: growthstandards (v.0.1.5), ontologyIndex (v.2.7), stats, NRejections (c.1.2.0). 4) Pathway analysis: Enrichr: https://maayanlab.cloud/Enrichr/.

For manuscripts utilizing custom algorithms or software that are central to the research but not yet described in published literature, software must be made available to editors and reviewers. We strongly encourage code deposition in a community repository (e.g. GitHub). See the Nature Portfolio guidelines for submitting code & software for further information.

Data

Policy information about availability of data

All manuscripts must include a data availability statement. This statement should provide the following information, where applicable:

- Accession codes, unique identifiers, or web links for publicly available datasets
- A description of any restrictions on data availability
- For clinical datasets or third party data, please ensure that the statement adheres to our policy

All genome-wide CNV association summary statistics are available at Zenodo (https://zenodo.org/record/7939126#.ZGK7yi-B29Y with DOI 10.5281/zenodo.7939126). Individual-level CNV data for epilepsy patients are available from the Epi25 Consortium (http://epi-25.org/) upon reasonable request and approved ethics protocol. Furthermore, raw data is deposited at dbGAP https://www.ncbi.nlm.nih.gov/projects/gap/cgi-bin/study.cgi?study_id=phs001551.v1.p1. All HPO-based phenome-wide summary statistics are available in Supplementary Table 6 of this manuscript. Fine-mapping results are available in Table 2 and Supplementary Tables 2-3 of this manuscript. The CNV data of the Neuropsychiatric cohort are described in the Supplementary Materials of Collins et al. (2022)97. They can be accessed from existing publications, public resources, or, upon request, from the authors of Collins et al. (2022)97 (see "Key resources table" and Table S2 in Collins et al.97). The CNV data reported by GeneDx and Indiana University clinical testing sites were not consented for public release. All datasets used in this study are detailed in Supplementary Table 1 of our manuscript."

Research involving human participants, their data, or biological material

Policy information about studies with human participants or human data. See also policy information about sex, gender (identity/presentation), and sexual orientation and race, ethnicity and racism.

Reporting on sex and gender	All findings apply to both sexes. Reported sex and sex estimated from the genotyping data were used to identify mislabeled samples during quality control. We did not perform sex-based analyses because there is no evidence of sex bias in the polygenic forms of epilepsy. According to local IRB requirements, patients or their legal guardians provided signed informed consent, including data sharing. Some centers collected samples for over 20 years, so the consent forms reflected standards at the time of collection. For Epi25 Consortium samples collected after January 25, 2015, consent forms required specific language according to the NIH Genomic Data Sharing Policy.
Reporting on race, ethnicity, or other socially relevant groupings	We did not construct or use socially relevant categorization.
Population characteristics	The study samples were divided into the following categories based on the clinical phenotype: 1) population level controls not enriched for epilepsy, 2) Individuals with clinically defined epilepsy following the International League Against Epilepsy (ILAE) classifications, 3) Individuals with seizure disorders, 4) individuals with a range of neuropsychiatric disorders, detailed in Supplementary Table 4: abnormality of the nervous system, abnormality of higher mental function, neurodevelopmental abnormality, autistic behavior, intellectual disability, behavioral abnormality, impairment in personality functioning, hyperactivity, bipolar affective disorder, schizophrenia, abnormal fear/anxiety-related behavior, sleep disturbance, abnormality of nervous system morphology, morphological abnormality of the central nervous system, abnormality of brain morphology, atrophy/Degeneration affecting the central nervous system, abnormality of central motor function, abnormality of movement, CNS hypermyelination, involuntary movements, abnormality of the peripheral nervous system, abnormal peripheral nervous system morphology, peripheral neuropathy. All individuals had SNP genotyping array data used to call copy number variants.
Recruitment	Individuals with epilepsy were recruited through the Epi25 Collaborative (http://epi-25.org/) an International multi-center epilepsy genetics research consortium. Ancestry-matched population controls (N=8,545) for the Epi25 arm of the study were recruited through 1) the Epi25 consortium, 2) a Broad Institute project on inflammatory bowel disease without reported epilepsy (part of the IBD Genetics Consortium IBDGC), 3) healthy individuals from the Genetics Personality Consortium (GPC), and 4) the THL Institute of Health and Welfare (subsample of FINRISK study). We only used summary statistics for the seizure and neuropsychiatric disorder arm of this study.
Ethics oversight	All research participants or their legal guardians provided written, informed consent using protocols approved by ethics committees at each study site (Supplementary Table 1).

Note that full information on the approval of the study protocol must also be provided in the manuscript.

Field-specific reporting

Please select the one below that is the best fit for your research. If you are not sure, read the appropriate sections before making your selection.

☒ Life sciences ☐ Behavioural & social sciences ☐ Ecological, evolutionary & environmental sciences

For a reference copy of the document with all sections, see nature.com/documents/nr-reporting-summary-flat.pdf

Life sciences study design

All studies must disclose on these points even when the disclosure is negative.

Sample size	In this study, we jointly analyzed all (currently) available European individuals with epilepsy and genotype intensity data for CNV analyses (N=16,109) with 10,590 individuals with seizure disorders. Our meta-analysis for 26,699 individuals with various types of seizure disorders against 492,324 population controls in the largest ever performed CNV-GWAS in any type of seizure disorder, including epilepsy. Our meta-analysis in seizure disorders replicated all three CNVs previously reported as associated with epilepsy at a genome-wide level in Niestroj et al. 2020, the previously largest CNV-GWAS study, and identified 25 novel loci with genome-wide significance for seizure disorders.
Data exclusions	Data (samples and called CNV) were excluded from the analyses only according to pre-established quality control (QC) parameters and following best practices standards for CNV-GWASs. We excluded individuals from the Epi25 cohort with a call rate <0.96, discordant sex status, and non-European ancestry.
Replication	All analysis parameters are given in the manuscript, and scripts to perform the association analyses are available online for reproducibility. No direct replication was attempted. However, our study replicated the genome-wide associations of Niestroj et al., 2020, the previous largest CNV-GWAS study in this type of disorder. We are, therefore, confident that most of the novel loci will be replicated in future studies.
Randomization	Randomization was irrelevant to this study's purpose; all samples were allocated to case/control groups based on the presence of any seizure disorder. Because we lumped a broad spectrum of seizure disorders in one group, it was not within the scope of this study to perform subphenotype analyses.
Blinding	The recruitment of cases and controls was blinded to the study protocol, purpose, and calling of the copy number variants.

Reporting for specific materials, systems and methods

We require information from authors about some types of materials, experimental systems and methods used in many studies. Here, indicate whether each material, system or method listed is relevant to your study. If you are not sure if a list item applies to your research, read the appropriate section before selecting a response.

Materials & experimental systems

n/a	Involved in the study	
☒	☐	Antibodies
☒	☐	Eukaryotic cell lines
☒	☐	Palaeontology and archaeology
☒	☐	Animals and other organisms
☒	☐	Clinical data
☒	☐	Dual use research of concern
☒	☐	Plants

Methods

n/a	Involved in the study	
☒	☐	ChIP-seq
☒	☐	Flow cytometry
☒	☐	MRI-based neuroimaging
